# Examining the Effect of Deep Learning-Based Image Reconstruction on Accelerating Shoulder Magnetic Resonance Imaging (MRI) and Its Impact on Image Quality

**DOI:** 10.7759/cureus.94561

**Published:** 2025-10-14

**Authors:** Jordan Zheng Ting Sim, Alexander Jiawei Yap, Yong Han Ting, Shu Wen Goh, Alvin Yong Quan Soon, Minju Cho, Sohyun Kim, Glen Chern Yue Ong

**Affiliations:** 1 Department of Diagnostic Radiology, Tan Tock Seng Hospital, Singapore, SGP; 2 Department of Diagnostic Radiology, Woodlands Health Campus, Singapore, SGP; 3 Department of Diagnostic Radiology, National University Hospital, Singapore, SGP; 4 Department of Research, AIRS Medical, Seoul, KOR

**Keywords:** artificial intelligence, deep learning, image quality, image reconstruction, shoulder mri

## Abstract

Background

Prolonged scan time remains the main obstacle to increasing magnetic resonance imaging (MRI) throughput. The advent of artificial intelligence brings forth opportunities to accelerate MRI examinations.

Purpose

This study compares the image quality of standard MRI versus accelerated MRI with deep learning-based image reconstruction (DLR) for shoulder MRI studies.

Materials and methods

Forty-nine subjects were prospectively enrolled and underwent both standard and accelerated axial proton density fat-saturated (PD FS) shoulder MRIs using a 1.5T scanner (Philips Ingenia 1.5T). Two blinded musculoskeletal radiologists independently evaluated paired datasets to assess the anatomic conspicuity of specific structures (labrum, rotator cuff footprint, cartilage, long head of the biceps tendon/rotator interval), artifacts, and overall image quality. A 5-point scale was employed, where 1 indicated the standard MRI was markedly superior and 5 indicated the accelerated MRI was markedly superior. The reduction in scan time was recorded; inter-reader variability was also analyzed.

Results

The DLR protocol reduced scan duration by 20.2% on average, shortening acquisition time from 184 seconds to 148 seconds. Mean scores for anatomic conspicuity ranged from 3.0 to 3.2, and mean scores for artifacts and overall image quality were 3.0 and 3.2, respectively. The Wilcoxon signed-rank test revealed statistically significant differences (p<0.001) for most categories, except for "Artifacts" as assessed by one reader. Inter-reader agreement was poor, with Cohen's kappa ranging from 0.086 to 0.183 and prevalence-adjusted bias-adjusted kappa (PABAK) scores ranging from 0.063 to 0.404.

Conclusion

DLR-based acceleration significantly reduces scan time while maintaining diagnostic image quality, presenting a clinically feasible and efficient solution for routine shoulder MRI.

## Introduction

The advent of artificial intelligence (AI) and deep learning (DL) marks a paradigm shift in radiology, encompassing areas such as image recognition [1], scan triaging [2], and, more recently, report generation via large language models (LLMs) [3]. This study explores the application of DL in an upstream radiology workflow, post-acquisition image reconstruction, specifically in magnetic resonance imaging (MRI).

Despite significant technological advancements, prolonged MRI scan times remain the main obstacle to increasing throughput and patient comfort. One of the key concepts of deep learning reconstruction (DLR) is to purposefully reduce image acquisition time, causing image quality degradation, while applying DL-based post-processing to generate images comparable to those obtained through standard protocols. Similar techniques can also improve images acquired at lower field strengths without extending scan duration, although this study focuses on the former.

DLR architectures can generally be categorized into image domain, k-space learning, and direct mapping types [4]. For this study, we utilized a Food and Drug Administration (FDA)-cleared, Conformité Européenne (CE)-certified, commercially available DLR software (SwiftMR, v3.0.3.0, AIRS Medical, Seoul, Korea) [5]. This is a Digital Imaging and Communications in Medicine (DICOM)-based algorithm, which means that it is MR vendor-neutral and works on all field strengths. Furthermore, as DICOM data is more readily available compared to raw k-space data, this approach simplifies implementation. Prior studies have demonstrated the effectiveness of DLR in reducing scan and room times while maintaining diagnostic integrity and image quality [6-8]. Studies specifically focusing on shoulder MRI have reported superior image quality, higher diagnostic confidence, and shorter scan durations with vendor-specific, k-space-based DLR [9-11]. However, the effect of the image-based DLR algorithm on shoulder MRIs has not yet been reported. Building upon these previous findings, this study aims to validate the clinical feasibility of DLR-accelerated MRI in a local context, focusing on its impact on shoulder MRI quality and workflow efficiency. Reducing scan time will lead to greater patient comfort and compliance, while increasing workflow efficiency enables throughput increase with better resource allocation.

## Materials and methods

Study design

This study was conducted at Tan Tock Seng Hospital, Singapore, after obtaining approval from the National Healthcare Group Domain Specific Review Board (DSRB) (approval number: 2023/00390-AMD0001), and the requirement for informed consent was waived. Forty-nine subjects (17 men, 32 women; mean age 61.7 years; range 22-87) who had shoulder MRI scans done between March 2023 and May 2023 were prospectively enrolled in this study, each undergoing both standard and accelerated axial proton density fat-saturated (PD FS) shoulder MRI scans on a 1.5 T scanner (Philips Ingenia 1.5 T). In musculoskeletal imaging, the shoulder often poses the greatest challenge in terms of image quality and interpretation due to its multi-directional (ball-in-socket) nature of anatomical structures and relative subtlety of pathologies (e.g., biceps pulley injury, superior labrum anterior to posterior (SLAP) tear, etc.). The study team made a deliberate attempt to test this DL tool on this challenging region. A formal sample size calculation was not performed for this study. The number of cases included was determined by the maximum permitted dataset size under the vendor-provided research license for the software. The remaining sequences were acquired using standard protocols to avoid excessively long total scan durations. The DLR sequence was made available to the MR operator on the console for quality assurance. 

**Figure 1:**
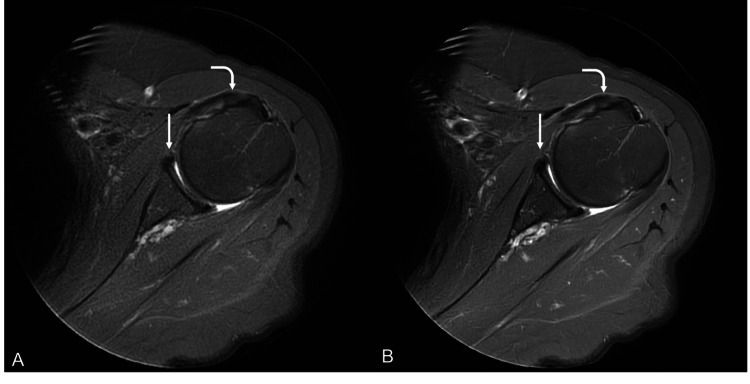
DLR-processed sequence (B) was scored as notably superior to the conventional sequence (A) by both readers. DLR-processed sequence (B) shows enhanced sharpness of the cartilage and labral outlines (straight arrows) while maintaining conspicuity of mild posterior labral fraying and subscapularis tendinosis (curved arrows) DLR: deep learning reconstruction

**Figure 2:**
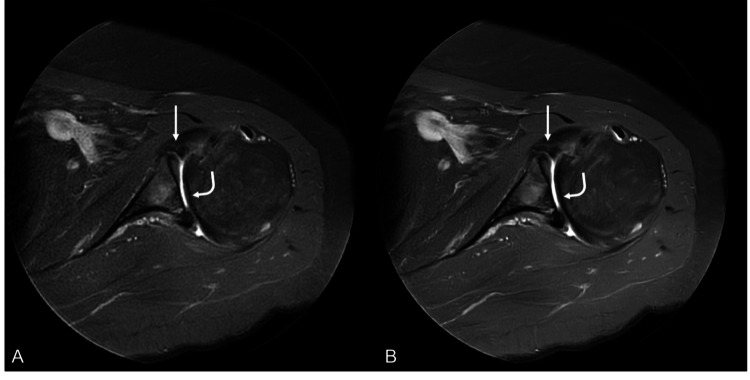
DLR-processed sequence (B) was scored as mildly superior to the conventional sequence (A) by both readers. Both sequences adequately depict a non-displaced anteroinferior labral tear (straight arrows) as well as glenohumeral cartilage wear (curved arrows), but the DLR-processed sequence exhibited improved sharpness and reduced noise DLR: deep learning reconstruction

**Figure 3:**
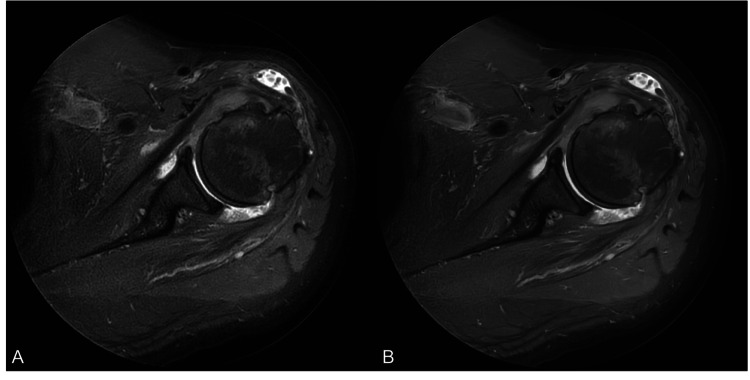
Conventional sequence (A) and DLR-processed sequence (B). Both readers indicated no perceivable difference. Findings of joint synovitis, subacromial subdeltoid bursitis, and severe subscapularis tendinosis are equally well-visualized on both sequences DLR: deep learning reconstruction

Two musculoskeletal radiologists (with three and 11 years of musculoskeletal MRI experience, respectively) independently reviewed images in a paired, side-by-side manner and evaluated for anatomic conspicuity of specific structures (labrum, rotator cuff footprint, cartilage, long head of biceps tendon/rotator interval), imaging artifacts, and overall image quality. The readers were blinded to the order of image acquisition, and the paired images were arranged in a random fashion. A 5-point scale was employed, where 1 indicated the standard MRI was markedly superior, 5 indicated the accelerated MRI was markedly superior, and 3 indicated no perceivable difference. This scale was adapted from a study by Bash et al. [7]. This directional preference scale allows direct head-to-head comparison of paired images, incorporates directionality (left vs. right) into the response, and retains a neutral midpoint for cases of no perceived difference. Additionally, by offering a different degree of superiority on either side, the scale can capture not just "which image is better" but also "how much better", which increases granularity compared to a binary forced choice. All images were reviewed in a randomized order, and the reduction in scan time for the accelerated sequence was recorded. Inter-reader variability was analyzed using multiple statistical approaches. Figures 1-2 represent examples in which the DLR-processed sequence was scored as notably and mildly superior to the conventional sequence. Figure 3 depicts a case in which no perceivable difference was noted.

DL reconstruction

The DLR software operates in the DICOM domain. It is a post-processing step that incorporates a deep neural network (DNN) based on the U-Net architecture [12]. The network features initial convolutional blocks with 64 output channels including four stages of down-sampling and up-sampling. The model's architecture is composed of 18 convolutional blocks with a kernel size of 3×3, along with layers for max pooling, bilinear up-sampling, and feature concatenation. These are followed by three additional convolutional layers with a kernel size of 1×1. Layer normalization was applied after each convolutional block, and dropout was not used. As a pre-processing step, the image size was resampled to 1024×1024 using Lanczos interpolation. This design was incorporated to improve the input MR images' spatial resolution while minimizing noise and ensuring structural accuracy.

The software addresses image quality by taking a multidimensional approach, considering various aspects of k-space sampling through the application of multidimensional degradation to raw k-space data during the model training phase. This process involved a combined application of noise addition and multiple undersampling patterns, including uniform, random, kmax, partial Fourier, and elliptical undersampling.

In addition to the above training inputs, auxiliary contextual data consisted of acquisition parameters associated with various k-space sampling patterns, and the expected noise reduction ratios for each training pair were also considered. These data enabled the network to handle the complexity of diverse degradation scenarios and were incorporated into the U-Net architecture by modifying the network with a dynamic modulation pathway, resulting in a Context-Enhanced U-Net (CE U-Net).

The training and validation sets consisted of approximately three million and 300,000 slices, respectively, with no data augmentation applied, encompassing a broad range of MRI sequences, anatomical regions, and acquisition parameters. The model was trained with a batch size of 4. L1 loss was used as the loss function to ensure the reconstructed images maintain the anatomical precision shown in the high-resolution images. Learning rate was set at 0.001 and was sequentially reduced by a factor of 0.1 after 10 epochs, followed by an additional 3 epochs of training. The model was optimized using Adam [13], leveraging four graphics processing units (GPU) (Tesla V100, NVIDIA Corporation, Santa Clara, California, United States) equipped with 32 GB of graphics memory each. The images for the study and those for the algorithm's training were mutually exclusive. Additional details regarding the model architecture, training, and validation process can be found in [5].

Statistical analysis

Inter-reader agreement was assessed using Cohen's kappa, percent agreement, and prevalence-adjusted bias-adjusted kappa (PABAK) to account for score skewness. To address potential inter-reader variability, scores were consolidated into three categories: 1 and 2 were grouped to represent lower ratings, 3 was retained as the neutral category, and 4 and 5 were grouped to represent higher ratings. This categorization was intended to simplify variability in the statistical analysis while preserving the interpretive granularity of the data. Score distributions for each criterion were analyzed, and paired ratings were compared using the Wilcoxon signed-rank test. This test evaluated whether differences between the original and DLR-processed images deviated significantly from a reference value of 3. All statistical analyses were performed using Python (v3.10). Values of p<0.05 were considered statistically significant. 

## Results

The implementation of DLR resulted in a significant reduction in scan time, with an average decrease of 20.2%. The acquisition time for the standard axial PD FS sequence was reduced from 184 seconds to 148 seconds, demonstrating the efficiency of the DLR protocol in streamlining imaging workflows.

**Figure 4:**
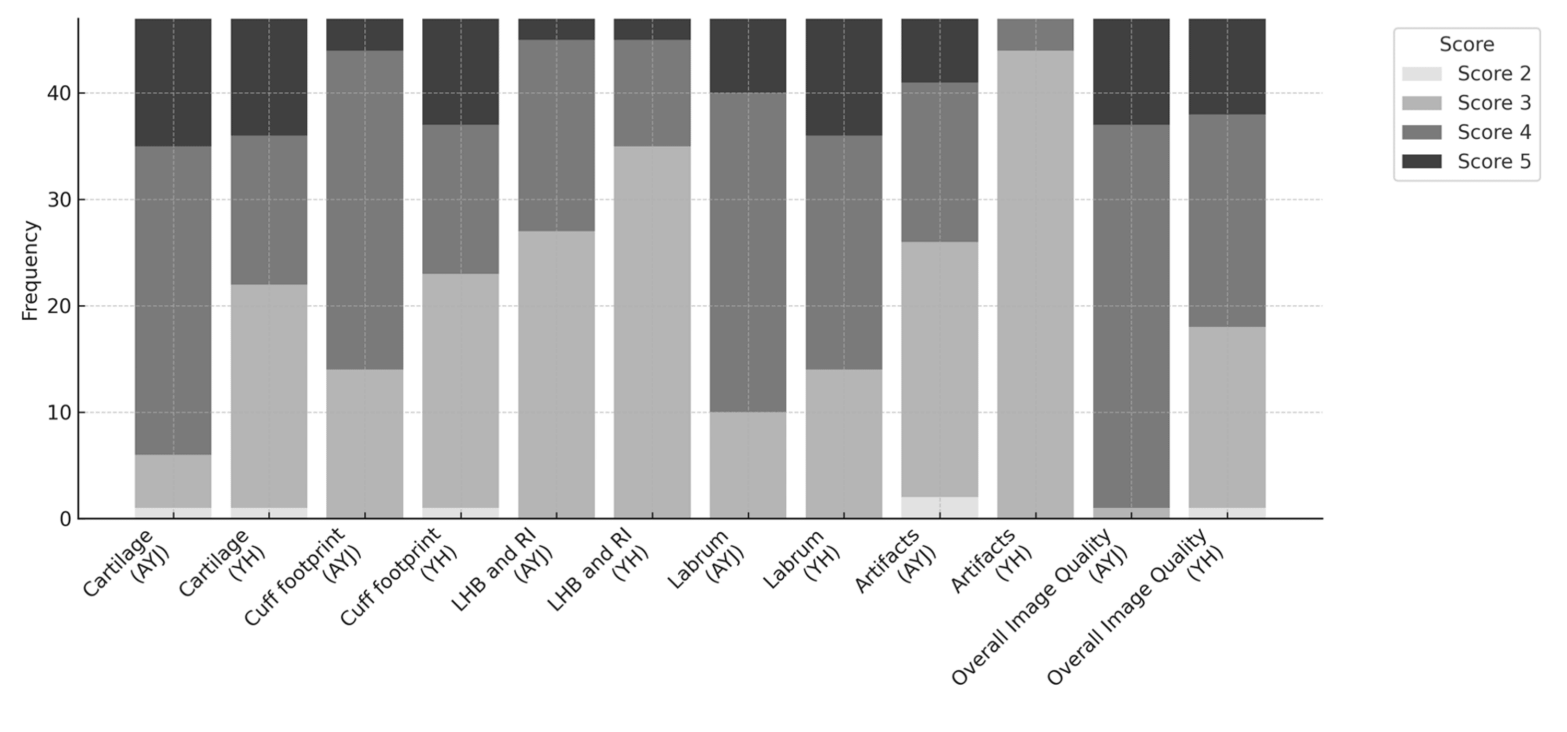
Score distributions for each image quality criterion and reader. Scores range from 1 to 5, indicating varying levels of image quality perception from the original image being superior to the DLR image being superior. Both readers predominantly rated scores of 3-4, with overall image quality and artifact reduction most frequently favoring the DLR images DLR: deep learning reconstruction

In the evaluation of anatomic conspicuity, the mean scores for labrum, rotator cuff footprint, cartilage, and long head of the biceps tendon/rotator interval were 3.0, 3.1, 3.2, and 3.1, respectively. Imaging artifacts and overall image quality were rated with mean scores of 3.0 and 3.2. These scores suggest that the DLR-accelerated images were largely comparable to, and in some cases slightly superior to, the standard protocol. Figure 4 plots the score distributions for each image quality criterion and reader.

**Figure 5:**
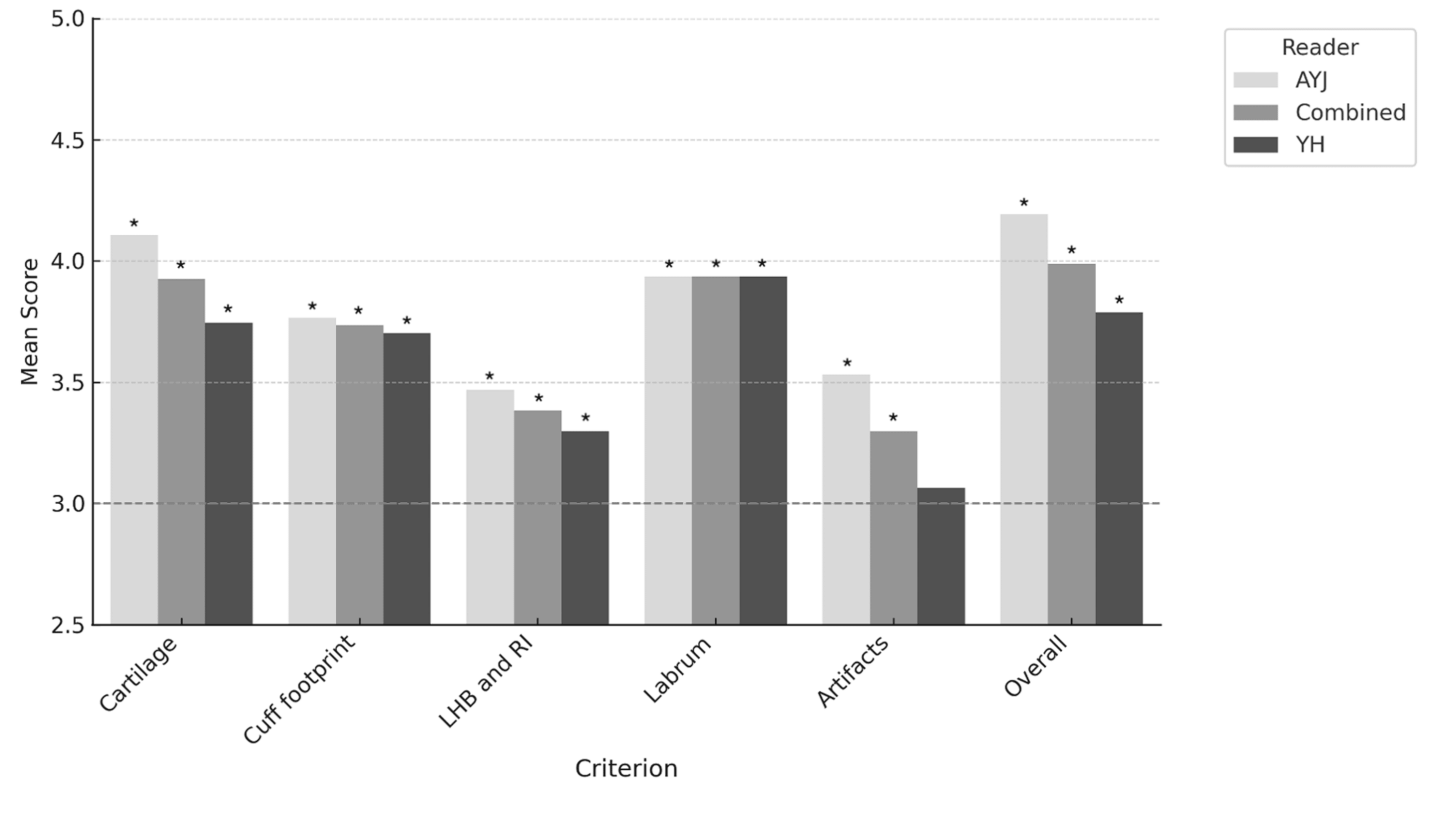
Wilcoxon test (reference score=3) comparing image quality scores from two radiologists (A.Y.J. and Y.H.). The chart presents mean scores across specific criteria, with asterisks (*) indicating significant differences from the reference (p<0.05). Scores above 3 reflect superior quality of DLR images. All mean scores exceeded 3, with all but one reaching statistical significance, most notably for the labrum and overall image quality DLR: deep learning reconstruction

Statistical analysis using the Wilcoxon signed-rank test showed significant differences between standard and reconstructed images across most categories (p<0.05). For the "Artifacts" category, a significant difference was observed for reader 1 (p<0.05), while no significant difference was detected for reader 2 (p=0.083). Combined analysis for this category demonstrated a statistically significant improvement (p<0.05), indicating that DLR effectively mitigated artifacts in the reconstructed images. Table 1 shows the mean and median scores of both readers. Figure 5 plots the results of the Wilcoxon test. 

**Table 1:** Mean and median of score given by both readers LHB: long head of biceps; RI: rotator interval

Metric	Reader 1 (A.Y.J.)	Reader 2 (Y.H.)
Anatomic conspicuity: labrum		
Mean (SD)	3.87 (1.12)	3.91 (1.18)
Median (Q1-Q3)	4.00 (3.00-4.00)	4.00 (3.00-4.00)
Anatomic conspicuity: cuff footprint		
Mean (SD)	3.74 (0.95)	3.69 (1.06)
Median (Q1-Q3)	3.00 (2.00-4.50)	3.00 (2.00-4.00)
Anatomic conspicuity: cartilage		
Mean (SD)	4.12 (1.29)	3.71 (1.10)
Median (Q1-Q3)	4.00 (3.00-4.00)	3.00 (3.00-4.00)
Anatomic conspicuity: LHB and RI		
Mean (SD)	3.45 (0.73)	3.31 (0.62)
Median (Q1-Q3)	3.00 (3.00-4.50)	3.00 (3.00-3.00)
Artifacts		
Mean (SD)	3.52 (0.91)	3.21 (0.25)
Median (Q1-Q3)	3.00 (3.00-4.00)	3.00 (3.00-3.00)
Overall image quality		
Mean (SD)	4.26 (1.27)	3.78 (1.09)
Median (Q1-Q3)	4.00 (2.00-4.00)	3.00 (2.50-4.00)

Inter-reader agreement, as measured by Cohen's kappa, indicated poor agreement across categories, with values ranging from 0.057 to 0.224. The "Artifacts" category demonstrated the lowest agreement (kappa=0.057), while the highest agreement was observed for the "Anatomic conspicuity of the Labrum" (kappa=0.224). PABAK scores reflected slightly better agreement, ranging from 0.064 for artifacts to 0.404 for labrum. These findings indicate moderate consistency in specific categories but emphasize variability in reader assessments, particularly for artifact evaluation.

## Discussion

This study examined the impact of DLR on the axial PD FS sequence in routine shoulder MRIs, demonstrating a notable reduction in scan times while maintaining or improving diagnostic image quality. Notably, Yang et al. previously reported greater scan and room time reductions in a large-scale, multi-centre study involving multiple MRI scanners and body parts [6]. A recent publication by Vosshenrich et al. concluded that DLR MRI examinations correlated well with arthroscopic findings with regard to internal derangement findings in the knee [14]. Specific to shoulder MRIs, Kaniewska et al. observed superior image quality and higher diagnostic confidence with accelerated PROPELLER sequences using DLR [9]. Similarly, Herrmann et al. concluded that the diagnostic performance of DLR-accelerated and standard turbo spin echo sequences was comparable [10]. These findings align with the results of this study and underscore the potential of DLR as a non-inferior alternative for reducing scan times without compromising image quality, akin to the principles of non-inferiority clinical trials [15]. By shortening scan durations, overall daily throughput can be improved, leading to more efficient resource utilization and fewer workflow bottlenecks [6,16]. For instance, MRI sessions traditionally allocated 30-minute slots can now be scheduled in 25- or even 20-minute intervals. Furthermore, such advancements could lead to improved patient compliance and comfort, particularly by reducing wait times and scan duration.

Both radiologists in this study acknowledged the effectiveness of DLR in artifact reduction and denoising, consistent with previous studies demonstrating the robust denoising capabilities of DLR methods [11]. However, the potential risk of "hallucinations" remains a critical consideration. A 2020 MRI reconstruction challenge revealed that many top-performing models introduced hallucinations, artificial structures not representative of actual anatomy, which were not captured by traditional image quality metrics [17]. Anecdotally, in our study, the expert reviewers also noted potentially hallucinatory findings in the cartilage and labrum. In one case, the reconstructed sequence revealed cartilage fraying that was not evident on the conventional sequence. In another, the DLR image made the patient's posterior labrum appear rounder compared to the conventional sequence. Both cases were subsequently reviewed by a third independent reader (G.O.) as part of an internal audit. The introduction of such artifacts, particularly when normal structures are misrepresented as pathological findings, is unacceptable. This highlights the importance of rigorous monitoring and validation frameworks to ensure the reliability of DLR-deployed systems.

Current commercially available MRI DLR software can be categorized into two main approaches: DICOM-based and k-space-based methods [5,18,19]. Each approach has its own set of advantages and limitations. K-space-based DLR methods are typically vendor-specific, integrating seamlessly with the same manufacturer's MRI systems provided that the scanner operates on a vendor-specified operating software platform version. DICOM-based methods are vendor-neutral, ensuring compatibility across the mixed fleet of scanners with different hardware configurations and software versions. However, the latter often requires an additional user interface post-deployment. Yang et al. reported that DICOM-based methods achieved greater reductions in scan and room times compared to k-space-based methods [6]. When integrating DLR into clinical workflows, careful consideration of each method's advantages and limitations is necessary to maximize efficiency while addressing operational challenges.

DLR is one of several techniques used to accelerate MRI acquisition. Methods such as parallel imaging, simultaneous multislice (SMS) acquisition, and compressed sensing (CS) have all contributed to faster MRI acquisition [20]. Additionally, synthetic MR contrast enhancement sequences, like synthetic STIR, have demonstrated comparable diagnostic image quality to conventional techniques [21,22]. Although these advancements and findings are promising, reductions in scan and room time may not directly lead to increased throughput in real-world scenarios. Further prospective studies are required to evaluate the actual effect of DLR reconstruction on throughput and to explore the technical and administrative challenges that hinder the full utilization of scan and room time savings. There is potential for substantial workflow improvements, including shorter examination slots, reduced appointment wait times, and enhanced patient throughput, ultimately benefiting clinical operations.

This study has limitations. First, it focused solely on a single sequence (axial PD FS) in one body part (shoulder). While other studies have validated their DLR techniques across entire shoulder MRI exams [9,10], concentrating on a single sequence allows for a larger sample size. This approach enables the evaluation of a broader range of pathologies and patient demographics while also avoiding prolonged examination times due to repeated sequences. Second, this study did not compare the performance of DLR with other commercially available software. Future work should explore the comparative performance of various DLR tools to identify optimal solutions for clinical adoption. Lastly, this study relied on relative qualitative metrics along with reader preferences and did not assess quantitative measures such as signal-to-noise ratio or contrast-to-noise ratio. While the algorithm's FDA clearance and established diagnostic quality provide reassurance, future studies should incorporate quantitative analyses to provide a more comprehensive evaluation. Another option would be to conduct the study with an arthroscopic correlate, which would act as the gold standard for diagnostic accuracy. Additionally, multi-institutional validation, along with quantitative metrics, will add strength to any clinically significant findings.

## Conclusions

This study demonstrates the clinical feasibility of using DLR to accelerate routine shoulder MRI using commercially available software. The findings indicate that DLR can meaningfully reduce scan times while maintaining diagnostic image quality and anatomical detail. While these results support the potential of DLR for improving workflow efficiency and patient experience, they should be interpreted in light of the study's limitations, including its modest sample size, focus on a single MRI sequence, and variable inter-reader agreement. A broader generalization of these findings will require further validation across diverse sequences, multiple institutions, and broader clinical contexts. Diligent monitoring is also essential to mitigate potential risks, such as reconstruction artifacts or hallucinations. Nonetheless, the present work provides preliminary evidence supporting DLR as a promising approach for MRI acceleration when applied with appropriate oversight.
